# Hepatic Insufficiency

**Published:** 1902-04

**Authors:** 


### Hepatic Insufficiency.

H. Richardson, *Philadelphia Medical Journal* of March 15th, argues that the bile salts are the only true chologogues. Only bile salts free from all impurities, such as cholesterin, should be used. Cases of hepatic colic, and the condition of incomplete bile elimination found in the insane, are readily relieved by administration of bile salts, and no return occurs while the treatment is continued. Many cases of glycosuria are hepatic in their origin and may be cured by a not very strict diet by the substitution of fats by starch and glycocholate of soda to assist in the assimilation. The bile salts being cumulative in their action and reabsorbed from the intestine, they need not be used over long periods of time, about two drams per month being taken in cases of hepatic colic.

